# Diagnostic performance of native T1 in grading histologically proven myocardial glycosphingolipid accumulation in Fabry disease

**DOI:** 10.1186/s13244-026-02262-z

**Published:** 2026-04-22

**Authors:** Nicola Galea, Livia Marchitelli, Giacomo Pambianchi, Lorenzo Dominici, Luca Conia, Maria Alfarano, Romina Verardo, Marco Francone, Cristina Chimenti, Andrea Frustaci, Carlo Catalano

**Affiliations:** 1 Department of Radiological, Oncological and Pathological Sciences, Policlinico Umberto I–Sapienza University of Rome, Rome, Italy; 2 Department of Clinical, Internal, Anesthesiology and Cardiovascular Sciences, La Sapienza University of Rome, Rome, Italy; 3 Cellular and Molecular Cardiology Lab, IRCCS L. Spallanzani, Rome, Italy; 4 Department of Medical Surgical Sciences and Translational Medicine, Sapienza–University of Rome, Radiology Unit–Sant’Andrea University Hospital, Rome, Italy

**Keywords:** Cardiac magnetic resonance, Endomyocardial biopsy, Fabry disease, Glycosphingolipids, T1 mapping

## Abstract

**Objective:**

Fabry disease cardiomyopathy (FD-CM) is a genetic disorder induced by glycosphingolipid accumulation (GSL-A) in cardiac cells, resulting in left ventricular hypertrophy (LVH) and contractile impairment. Native T1 (nT1) mapping by cardiac magnetic resonance (CMR) effectively detects myocardial GSL-A in patients with FD-CM. However, the association between nT1 values and GSL-A severity has not been histologically confirmed. This study aims to assess the capability of nT1 to classify GSL-A burden, using endomyocardial biopsy (EMB) as the reference, and to identify the best matching site for nT1 measurement.

**Materials and methods:**

Forty FD-CM subjects undergone CMR and EMB were classified into 4 groups according to GSL-A severity. CMRs were performed using a comprehensive protocol including nT1 and T2 mapping sequences. Global, per-plane, and septal segmental myocardial nT1 and T2 values (excluding late gadolinium enhanced areas) were compared among groups. Correlations between nT1 values, GSL-A and LVH were explored. Statistical analyses used distribution-appropriate tests, including group comparisons, correlation, and ROC analyses.

**Results:**

Significant nT1 differences emerged among groups at all measurement sites, with significant negative correlations with GSL-A, particularly in AHA segment 9, the mid-interventricular septum region of interest and midventricular plane (r = −0.741; −0.716; −0.715; *p* < 0.001). nT1 measured at these locations demonstrated excellent diagnostic accuracy in identifying severe GSL-A (area under the curve: 0.860, 0.903, and 0.895, respectively).

**Conclusion:**

Myocardial nT1 values strongly correlate with GSL-A, with midventricular septum measurements providing the best agreement. If supported by further studies, CMR could act as a noninvasive tool for disease staging and risk stratification, helping to identify patients in advanced, higher-risk stages.

**Critical relevance statement:**

Native T1 mapping correlates closely with histological glycosphingolipid accumulation in Fabry cardiomyopathy. This noninvasive biomarker may support early disease stratification and guide timely therapeutic intervention.

**Key Points:**

- Native T1 values are a valid biomarker of glycosphingolipid accumulation in myocardial tissue and correlate with accumulation degree.
- Midventricular interventricular septum is the preferred site for nT1 measurement, showing the best agreement with glycosphingolipid accumulation at endomyocardial biopsy.
- The use of native T1 in the setting of suspected cardiac Fabry disease will help to stratify disease severity, without the intrinsic risk of endomyocardial biopsy.

**Graphical Abstract:**

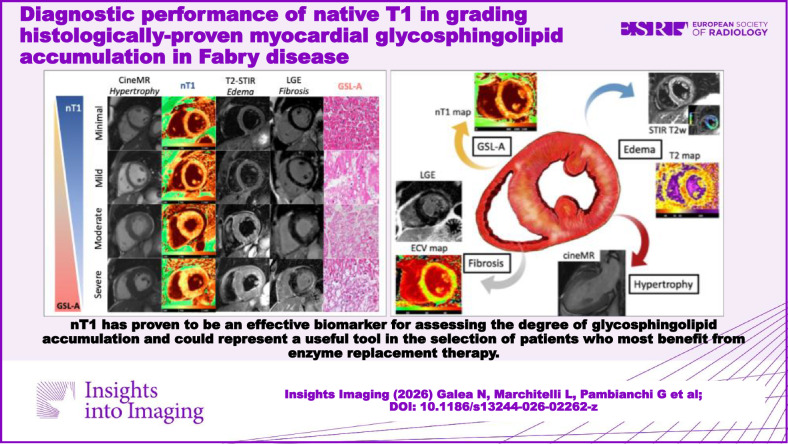

## Introduction

Fabry disease (FD) is a rare, X-linked lysosomal storage disorder caused by a deficiency of α-galactosidase A and resulting in intracellular glycosphingolipid accumulation (GSL-A). Cardiac involvement represents one of the major causes of morbidity and mortality in FD. Clinical manifestations of Fabry Disease Cardiomyopathy (FD-CM) are heterogeneous, ranging from left ventricular hypertrophy (LVH) to arrhythmias, conduction disturbances, valvular and ischemic heart diseases [1]. Histologically, myocardial GSL-A causes cardiomyocyte hypertrophy, arteriolar intima-media thickening and impairment of the conduction system [2, 3]. In cardiomyocytes, GSL-A compromises mitochondrial energy production, leading to cell apoptosis and necrosis [4]. Additionally, the release of lipidic antigens triggers the recruitment of inflammatory infiltrates and subsequent myocardial fibrosis [1, 5, 6].

Endomyocardial biopsy (EMB) remains the gold standard for the diagnosis of cardiomyocyte GSL-A, and plays a pivotal role in cases of unexplained severe LVH, or for monitoring response to enzyme replacement therapy (ERT). However, EMB is invasive, costly and associated with procedural risks [7]. Cardiac magnetic resonance (CMR) imaging, particularly by using T1 mapping sequence, has emerged as a valid noninvasive tool for assessing FD-CM, providing a comprehensive evaluation of ventricular morphology, function and myocardial tissue characteristics [8, 9]. Myocardial native T1 (nT1) values decrease when glycosphingolipids accumulate within cardiomyocytes, even preceding the LVH and contractile dysfunction [10].

Although nT1 is emerging as a surrogate marker of myocardial GSL-A in FD patients [11–13], its relationship with the histologically assessed glycosphingolipid burden remains unvalidated. This missing correlation is a key gap and should be addressed to determine whether nT1 can reliably reflect or stratify the severity of myocardial involvement. Furthermore, growing evidence indicates the presence of subclinical myocardial inflammation in FD-CM, which is associated with elevated troponin levels and worse clinical outcomes [14, 15] and may also affect T1 values. The aim of our study was to investigate the capability of nT1 values to differentiate degrees of myocardial GSL-A in FD patients and to identify the most representative myocardial site for nT1 measurement, using EMB as the reference standard.

## Materials and methods

### Study population

This was a single-center observational retrospective study on a cohort of 40 naïve FD patients, diagnosed by α-GalA activity and GLA mutation analyses, who underwent CMR examination and EMB.

Clinical indications for EMB were (1) resistant angina with no coronary artery obstruction or (2) unexplained ventricular arrhythmias in mutation carriers and (3) unexplained severe LVH.

Exclusion criteria were (1) history of other known cardiovascular (CV) diseases or electrocardiogram (EKG) findings consistent with previous myocardial infarction (MI); (2) evidence of systemic autoimmune disorders, sarcoidosis, amyloidosis; (3) poor CMR image quality; (4) detection of late gadolinium enhancement (LGE) with an ischemic distribution pattern.

The study selection flowchart is summarized in Supplementary data online (Fig. S1).

The study has been approved by the local ethical committee (Comitato Etico Territoriale Lazio Area 1; code n 301\2025; date of approval: 3/26/2025), and written informed consent was obtained from all participants.

To establish scanner-specific reference ranges for nT1, T2 and ECV, a cohort of 100 controls of both sexes and across a wide age range was analyzed. All showed no myocardial disease based on clinical data, echocardiography, or CMR. This group defined the normal myocardial tissue values for the scanner used, enabling accurate detection of abnormalities in FD patients. More detailed information is provided in the Supplementary data online (Appendix).

For each parameter (nT1, T2 and ECV), values from controls were used to derive normal thresholds based on the 95th percentile, accounting for physiological variability. These scanner-specific cutoffs were then applied to the FD cohort to distinguish normal from abnormal myocardial characteristics.

### CMR scanning protocol

All CMR examinations were performed on a 1.5-T scanner (Magnetom Avanto; Siemens Healthcare) equipped with a 16-channel phased-array surface coil. Intravenous administration of contrast medium (gadobutrol, Gadovist; Bayer Healthcare) was performed at a dose of 0.15 mmol/kg and a flow rate of 2.0 mL/s.

The standardized CMR protocol included cine imaging for cardiac function assessment, T1 and T2 mapping sequences for tissue characterization, and late gadolinium enhancement (LGE) imaging for the detection of focal fibrosis. Specifically, the following sequences were acquired: black-blood T2-weighted short-tau inversion recovery (T2w-STIR), T2-prepared True-FISP (for T2 mapping), modified Look-Locker inversion recovery (for T1 mapping, acquired before and 15–20 min after contrast injection to calculate ECV), balanced steady-state free precession cine-MRI, and contrast-enhanced inversion-recovery T1-weighted imaging (10–15 min after contrast administration) for LGE assessment.

Further details of the acquisition parameters are provided in the Supplementary Appendix.

### CMR images post-processing and analysis

CMR images were analyzed by two experienced cardiovascular radiologists with 6 (L.M.) and 17 (N.G.) years of experience, in consensus and blinded to EMB results, using a dedicated software (cmr42© v.5.3.0, Circle Cardiovascular Imaging Inc.).

Myocardial nT1, T2 and ECV values were evaluated by semiautomatically contouring subepicardial and subendocardial layers on respective maps, carefully excluding the epicardial fat, ventricular cavity and LGE areas (Fig. S2). Segmentation was performed automatically following the 17 American Heart Association (AHA) segmentation method [16].

To evaluate inter-observer variability, the two observers independently measured nT1 values from 20 FD patients (L.M. and N.G.). One of the two observers (L.M.) measured nT1 values twice, to assess intra-observer variability. The time delay between two reads for intra-observer variability was 2 weeks. Balanced steady-state free precession cine-MRI (CineMR) images have been analyzed to compute LV and right ventricular volumes, LV myocardial mass (LVMM) and maximal wall thickness (MWT), indexed for body surface area (BSA).

In each patient, nT1, T2 and ECV values were calculated globally, per plane (basal and midventricular planes) and per segment, according to AHA classification.

**Fig. 1:**
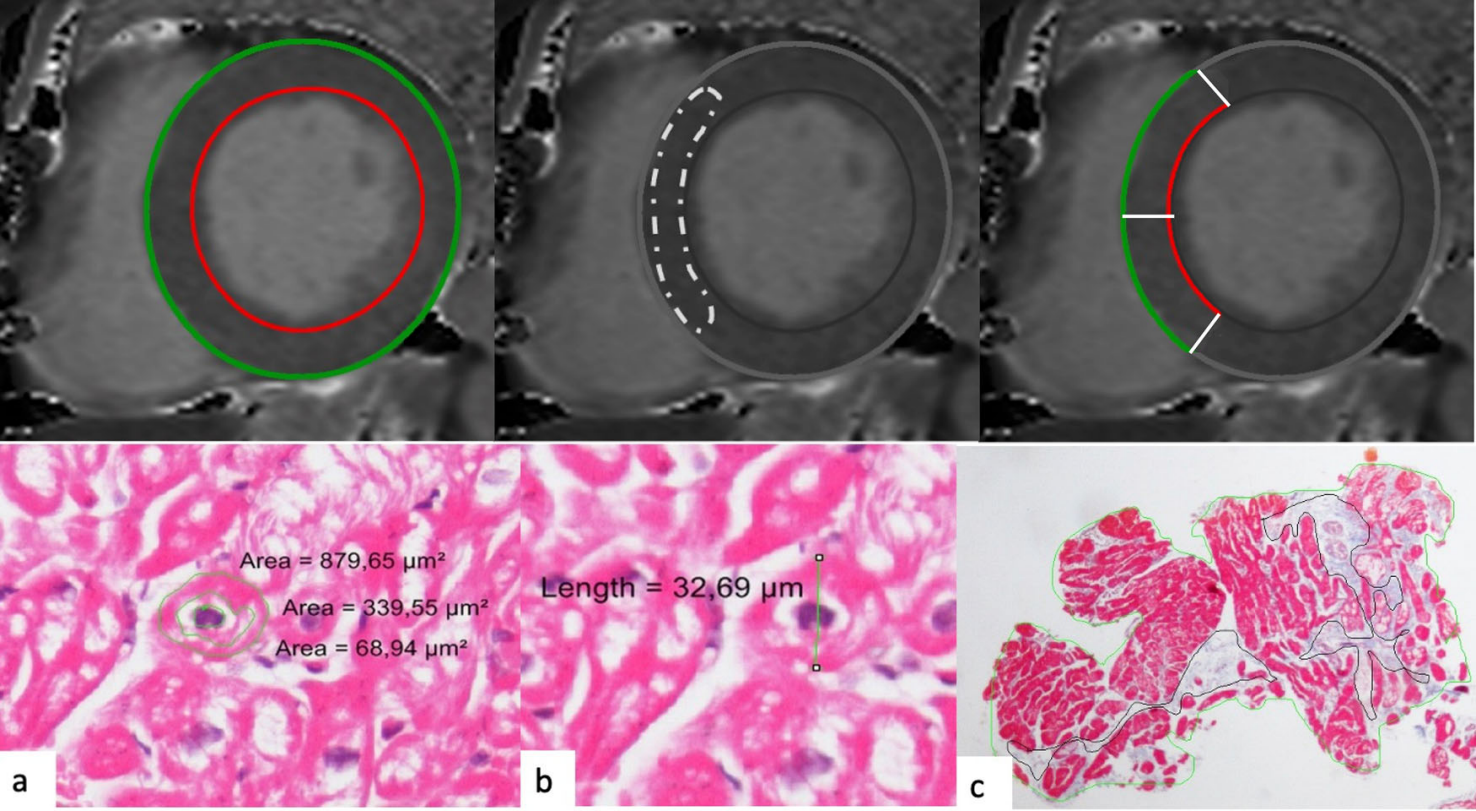
nT1 values assessment and morphometric analysis of EMB specimens. Upper row: nT1 values were calculated per plane (on the left, as the area included between epicardial (green) and endocardial (red) contours), in a region of interest drawn within the interventricular septum (in the center image) and per segment (basal anteroseptal (segment 2) and inferoseptal (segment 3)). Lower row: Assessment of cell area occupied by GSL vacuoles (**a**), cardiomyocytes diameter measurement (**b**) and fibrosis amount calculation (**c**)

Additionally, nT1 and T2 values of 2 regions of interest (ROI) placed within the interventricular septum (IVS) at the basal and midventricular segment (b- and mIVS ROI) were also measured (Fig. 1, upper row).

LGE and STIR images were considered positive according to current standards [17, 18].

The extent of LGE was quantified using the full-width at half-maximum method. For each subject, both the absolute LGE mass (g) and the percentage of LGE relative to total left ventricular myocardial mass were calculated.

The 95th percentile values in the control group served as thresholds for abnormalities, with T1 ranging from 970 to 1027 ms, T2 and ECV being less than 49.9 ms and 29.5%, respectively.

### Histology and electron microscopy

All patients underwent EMB, with no complications observed during or after the procedure.

Biopsies (4–5 samples from each ventricular chamber) were performed in the septal mid-apical region of the left or both ventricles.

All biopsy samples underwent a morphometric test (Fig. 1, lower row), which, as previously described [19], included (1) percentage cell area occupied by storage vacuoles, (2) evaluation of the diameter of the cardiomyocyte and (3) percentage of myocardial fibrosis in the specimen.

For each patient, all biopsy samples were analyzed separately, and quantitative morphometric data were averaged to obtain the final histological value. The overall histological diagnosis was based on the concordant findings among all samples.

Patients were stratified into four groups according to the degree of cardiomyocyte vacuolization, by dividing into quartiles: Group 1 or minimal (GSL-A: ≤ 25%), Group 2 or mild (26–35%), Group 3 or moderate (36–44%) and Group 4 or severe (≥ 45%).

Inflammatory infiltrates were defined by the presence of ≥ 7 lymphocytes per 2 mm², positive for CD45Ro immunostaining, in association with necrosis of adjacent myocytes, which was considered diagnostic of active inflammation overlapping Fabry cardiomyopathy.

CMR sequences and EMB procedure details are reported in the Supplementary Appendix.

### Statistical analysis

Data are presented as counts and percentages for categorical variables and as means ± standard deviation (SD) or medians with interquartile ranges (IQR) for continuous variables, as appropriate. The Kolmogorov–Smirnov and Shapiro–Wilk tests were used to assess the normality of data distribution. Depending on the results of these tests, parametric or non-parametric methods were applied.

Comparisons between categorical variables were performed using the Chi-squared (χ²) test. For continuous variables, one-way ANOVA was used for normally distributed data and the Kruskal–Wallis test for non-normally distributed data. When significant differences were found, post hoc subgroup analyses were performed using Fisher’s least significant difference and Tukey’s honest significant difference tests, which were selected to explore pairwise group differences while maintaining statistical power.

Receiver operating characteristic (ROC) curve analysis was performed to assess the diagnostic accuracy of nT1 in predicting the severity of GSL-A. The optimal cutoff value was determined using Youden’s index, maximizing the sum of sensitivity and specificity.

Linear correlations between continuous variables were evaluated using the Pearson correlation coefficient. Intra- and inter-observer reproducibility of nT1 measurements was assessed using the intraclass correlation coefficient (ICC).

All tests were two-tailed, and a *p*-value < 0.05 was considered statistically significant. Given the exploratory design and limited sample size, no formal correction for multiple comparisons was applied; however, results were interpreted with caution to avoid type I error inflation.

Statistical analyses were performed using SPSS software version 27.0 (IBM, Armonk).

## Results

### Study population

From April 2014 to October 2020, a total of 42 patients were recruited. Two patients were excluded due to insufficient image quality. The final cohort consisted of 40 naïve FD patients (M: 15; F: 25), with a mean age of 48 ± 15 years.

Demographic, clinical and histological characteristics are shown in Table 1.

**Table 1:** Demographic, clinical and histopathological characteristics

	FD patients (*N* = 40)	Group 1 (*N* = 10)	Group 2 (*N* = 10)	Group 3 (*N* = 10)	Group 4 (*N* = 10)	*p*-value
**Clinical features**						
Age (years), mean ± SD	48.2 ± 15.5	38.5 ± 18.3	50.2 ± 14.5	47.4 ± 14.1	56.6 ± 10.4	0.063
Male sex, *n* (%)	15 (37.5)	3 (30)	3 (30)	3 (30)	6 (60)	0.410
BSA (m^2^), mean ± SD	1.7 ± 0.2	1.7 ± 0.2	1.7 ± 0.2	1.8 ± 0.2	1.8 ± 0.3	0.328
Hs-TnT (mcg/dL), mean ± SD	0.05 ± 0.03	0.07 ± 0.02	0.05 ± 0.03	0.03 ± 0.03	0.04 ± 0.04	0.012*
CV risk factors, *n* (%)	27 (67.5)	6 (60)	7 (70)	6 (60)	8 (80)	0.740
Hypertension	18 (45)	3 (30)	5 (50)	3 (30)	7 (70)	0.217
Dyslipidemia	9 (22.5)	/	3 (30)	1 (10)	5 (50)	0.022*
Overweight	3 (7.5)	/	2 (20)	1 (10)	/	0.265
Family history	1 (2.5)	1 (10)	/	/	/	0.380
Smoke habit	1 (2.5)	/	1 (10)	/	/	0.380
Symptoms, *n* (%)	40 (100)	10 (100)	10 (100)	10 (100)	10 (100)	-
Palpitation	32 (80)	7 (70)	10 (10)	6 (60)	9 (90)	0.100
Chest discomfort	10 (25)	4 (40)	2 (20)	3 (30)	1 (10)	0.446
Dyspnea	4 (10)	/	/	2 (20)	2 (20)	0.217
Angina	4 (10)	2 (20)	/	2 (20)	/	0.217
EKG abnormalities, *n* (%)	37 (92.5)	7 (70)	10 (100)	10 (100)	10 (100)	0.021*
Short PR	15 (37.5)	2 (20)	6 (60)	1 (10)	6 (60)	0.031*
LVH signs	13 (32.5)	/	3 (30)	2 (20)	8 (80)	0.001*
LBBB	6 (15)	/	2 (20)	3 (30)	1 (10)	0.270
Other	4 (10)	2 (20)	/	2 (20)	/	0.217
**Histological features**						
Myocardiocytes diameters (µm), mean ± SD	29.6 ± 12	22.3 ± 6	28.6 ± 11.3	27. ± 9.8	39.8 ± 13.4	0.050
Storage vacuoles accumulation (%), mean ± SD	35.5 ± 11.9	20.5 ± 5.1	30.9 ± 2.7	39.8 ± 2.4	51 ± 4.5	< 0.001**
Myocardial fibrosis accumulation (%), median [IQR]	1 [1–3]	1 [1]	1.5 [1–3.3]	1.5 [1–3]	2 [2–4.5]	0.017*
Inflammatory infiltrates, *n* (%)	20 (50)	3 (30)	6 (60)	3 (30)	8 (80)	0.066

*FD* Fabry disease, *SD* standard deviation, *BSA* body surface area, *EKG* electrocardiogram, *Hs-TnT* high-sensitive Troponine T, *CV* cardiovascular, *LVH* left ventricle hypertrophy, *LBBB* left bundle branch block

* *p* < 0.05, ** *p* < 0.001

### CMR data

Only 4/40 patients presented LVEF reduction, and 7/40 patients showed increased left ventricle end diastolic volume (LVEDV)/BSA volume.

LVH was found in 18/40 patients: mild LVH (11/12 mm < MWT < 15 mm) was found in 9 (22.5%) patients, moderate LVH (15 mm ≤ MWT < 18 mm) in 5 (12.5%) patients and severe LVH (MWT ≥ 18 mm) in 4 (10%) patients.

CMR findings are displayed in Tables 2 and 3.

**Table 2:** CMR parameters

CMR features	Total *n*: 40	Group 1 Minimal GSL-A *n*: 10	Group 2 Mild GSL-A *n*: 10	Group 3 Moderate GSL-A *n*: 10	Group 4 Severe GSL-A *n*: 10	*p*-value
LVEDV/BSA (mL/m^2^), mean ± SD	78.5 ± 22.2	68.4 ± 20.7	74.7 ± 12.7	74.1 ± 16.3	96.9 ± 27.5	0.016*
LVESV/BSA (mL/m^2^), mean ± SD	26 ± 10.6	24.3 ± 10.2	23.3 ± 8.5	23.8 ± 9.6	33.5 ± 12.4	0.118
LVEF (%), mean ± SD	60.8 ± 9.2	61.5 ± 5.4	65.0 ± 6.9	62.7 ± 6.4	53.9 ± 13.1	0.034*
LVMM/BSA (g/m^2^), median [IQR]	51.9 [45.1–81.4]	45.6 [35.3–52.6]	48.8 [41.9–76.8]	54.1 [45.7–66.0]	93.3 [80.1–113.6]	0.026*
MWT (mm), median [IQR]	11.2 [9.6–14.4]	9.90 [8.9–11.6]	10.20 [8.8–13.9]	10.3 [10.15–14.4]	14.7 [13.0–17.9]	0.005*
RVEDV/BSA (mL/m^2^), mean ± SD	81.4 ± 19.1	75.8 ± 24.2	78.4 ± 13.1	80.0 ± 17.8	91.5 ± 18.9	0.275
RVESV/BSA (mL/m^2^) mean ± SD	34.3 ± 9.8	32.6 ± 10.7	30.4 ± 6.4	34.8 ± 11.3	39.5 ± 9.1	0.195
RVEF (%) mean ± SD	56.0 ± 5.6	54.5 ± 4.4	58.0 ± 4.0	56.1 ± 6.9	55.3 ± 6.9	0.565
Edema on T2w images/map, *n* (%)	13 (32.5)	1 (10)	1 (10)	4 (40)	7 (70)	0.010*
LGE presence, *n* (%)	17 (42.5)	2 (20)	4 (40)	3 (30)	8 (80)	0.037*
LGE quantitative, g	8.16 ± 13.3	3.4 ± 7.3	3.4 ± 5.2	6.0 ± 10.2	19.8 ± 19.5	0.010*
LGE quantitative, %	6.1 ± 8.5	4.3 ± 9.2	3.1 ± 5.4	4.5 ± 7.3	12.4 ± 9.3	0.049*

*LV* left ventricle, *BSA* body surface area, *EDV* end diastolic volume, *ESV* end systolic volume, *EF* ejection fraction, *LVM* left ventricle myocardial mass, *MWT* maximum wall thickness, *RV* right ventricle, *ECV* extracellular volume, *T2w* T2-weighted, *LGE* late gadolinium enhancement

* *p* < 0.05

**Table 3:** Native T1 values according to histopathological severity group

Parameter	Overall *n*: 40	Group 1 Minimal GSL-A *n*: 10	Group 2 Mild GSL-A *n*: 10	Group 3 Moderate GSL-A *n*: 10	Group 4 Severe GSL-A *n*: 10	*p*-value
nT1 global (ms), mean ± SD	955.8 ± 50.7	991.0 ± 33.0	962.4 ± 33.7	963.9 ± 48.3	906.0 ± 48.0	< 0.001**
nT1 basal plane (ms), mean ± SD	973 [934.5–1006]	1005 [972.1–1015.1]	995.5 [960.75–1016.8]	949 [942.25–1005.3]	911.5 [863.5–962.3]	0.009*
nT1 mid plane (ms), mean ± SD	949.0 ± 51.1	999.2 ± 39.7	950.1 ± 31.3	946.6 ± 42.8	900.2 ± 38.2	< 0.001**
nT1 AHA segment 2 (ms), mean ± SD	968.4 [922.76–1007.68]	1007.8 [967.1–1015.1]	979.9 [921–998.5]	956.65 [940.55–1007]	912.2 [890.4–938]	0.010*
nT1 AHA segment 3 (ms), mean ± SD	965.1 ± 61.6	1016.0 ± 51.8	963.0 ± 49.8	974.5 ± 45.2	906.7 ± 49.6	< 0.001**
nT1 AHA segment 8 (ms), mean ± SD	950.9 ± 61	1003.2 ± 43.8	959.4 ± 44.4	949.2 ± 61.8	891.9 ± 38.0	< 0.001**
nT1 AHA segment 9 (ms), mean ± SD	950.5 ± 57.3	1008.1 ± 35.2	952.1 ± 43.5	948.1 ± 50.0	893.8 ± 35.9	< 0.001**
nT1 bIVS ROI (ms), mean ± SD	973.5 [913.5–998.75]	991 [968.8–1017.5]	987.15 [909.8–1004.5]	938.5 [925.8–1004.8]	907.5 [864.8–938.8]	0.012*
nT1 mIVS ROI (ms), mean ± SD	940.6 ± 55.5	989.6 ± 44.3	946.8 ± 39.7	942.8 ± 44.6	883.3 ± 37.8	< 0.001**
T2 global (ms), mean ± SD	48 ± 2.6	47.3 ± 1.5	46.7 ± 1.5	48.1 ± 1.8	50.0 ± 3.3	0.020*
T2 basal plane (ms), mean ± SD	47.6 ± 2.6	47.2 ± 1.6	45.8 ± 2.1	47.7 ± 2.0	49.8 ± 3.1	0.003*
T2 mid plane (ms), mean ± SD	48.4 ± 3.1	47.4 ± 3.9	47.7 ± 1.9	48.4 ± 2.0	50.1 ± 3.8	0.209
T2 AHA segment 2 (ms), mean ± SD	46.9 ± 3.4	47.6 ± 2.3	47.8 ± 3.0	45.1 ± 3.6	46.9 ± 4.2	0.275
T2 AHA segment 3 (ms), mean ± SD	46.8 ± 2.9	44.7 ± 1.9	46.1 ± 2.2	48.4 ± 3.0	48.2 ± 3.0	0.007*
T2 AHA segment 8 (ms), mean ± SD	48.6 ± 3.9	47.7 ± 4.7	48.4 ± 3.3	48.3 ± 3.6	50.1 ± 4.0	0.557
T2 AHA segment 9 (ms), mean ± SD	46.9 ± 3.0	45.5 ± 2.5	45.4 ± 2.5	47.7 ± 2.2	49.1 ± 3.2	0.007*
T2 bIVS ROI (ms), mean ± SD	46.9 ± 2.7	46.0 ± 2.0	46.6 ± 2.0	47.0 ± 2.8	47.0 ± 3.7	0.399
T2 mIVS ROI (ms), mean ± SD	46.9 ± 3.6	46.0 ± 4.0	46.3 ± 3.1	47.3 ± 3.8	48.2 ± 3.4	0.505
Global ECV (%), mean ± SD	27.4 ± 5.2	25.8 ± 3.0	25.4 ± 4.1	27.7 ± 6.7	30.7 ± 5.1	0.079

*nT1* native T1, *SD* standard deviation, *AHA* American Heart Association, *IVS* interventricular septum, *ROI* region of interest

* *p* < 0.05, ** *p* < 0.001

Abnormal nT1, ECV and T2 values were found in 30/40 (75%), 21/40 (52.5%) and 11/40 (27.5%) patients, respectively, in at least one measurement site.

**Fig. 2:**
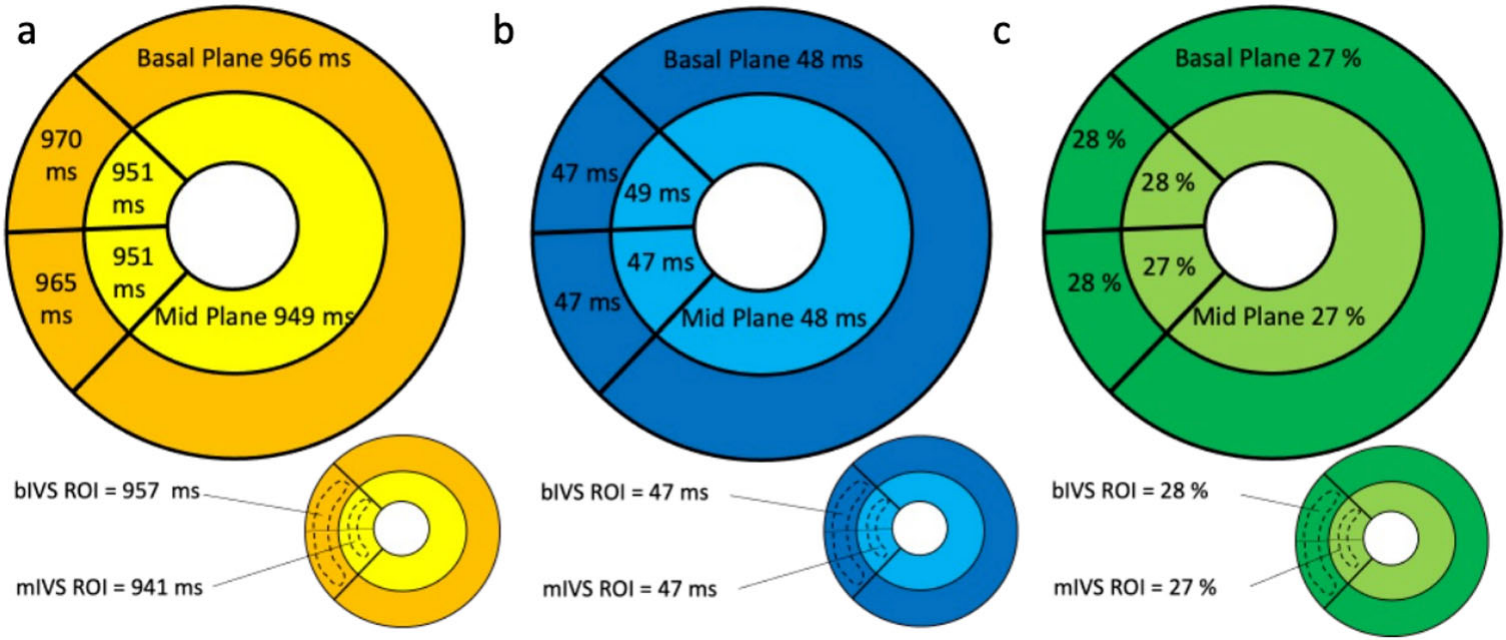
Bullseye view of basal and midventricular planes. Global nT1 (**a**), T2 (**b**) and ECV (**c**) mean values and inferoseptal and anteroseptal nT1 (**a**), T2 (**b**) and ECV (**c**) mean values for all patients

nT1,T2 and ECV values per plane and per segment of the overall patient population are shown in Fig. 2.

nT1 values showed excellent intra-reader reproducibility (ICC: 0.91, *p* < 0.001) and inter-reader reproducibility (ICC: 0.85, *p* = 0.001) without any significant systematic bias.

### nT1 values according to LVH severity

All patients with moderate-to-severe LVH showed decreased nT1 values in at least one measurement site, while the prevalence for pre-hypertrophic and mild LVH patients was 14/22 (63.6%) and 7/9 (77.8%), respectively.

**Fig. 3:**
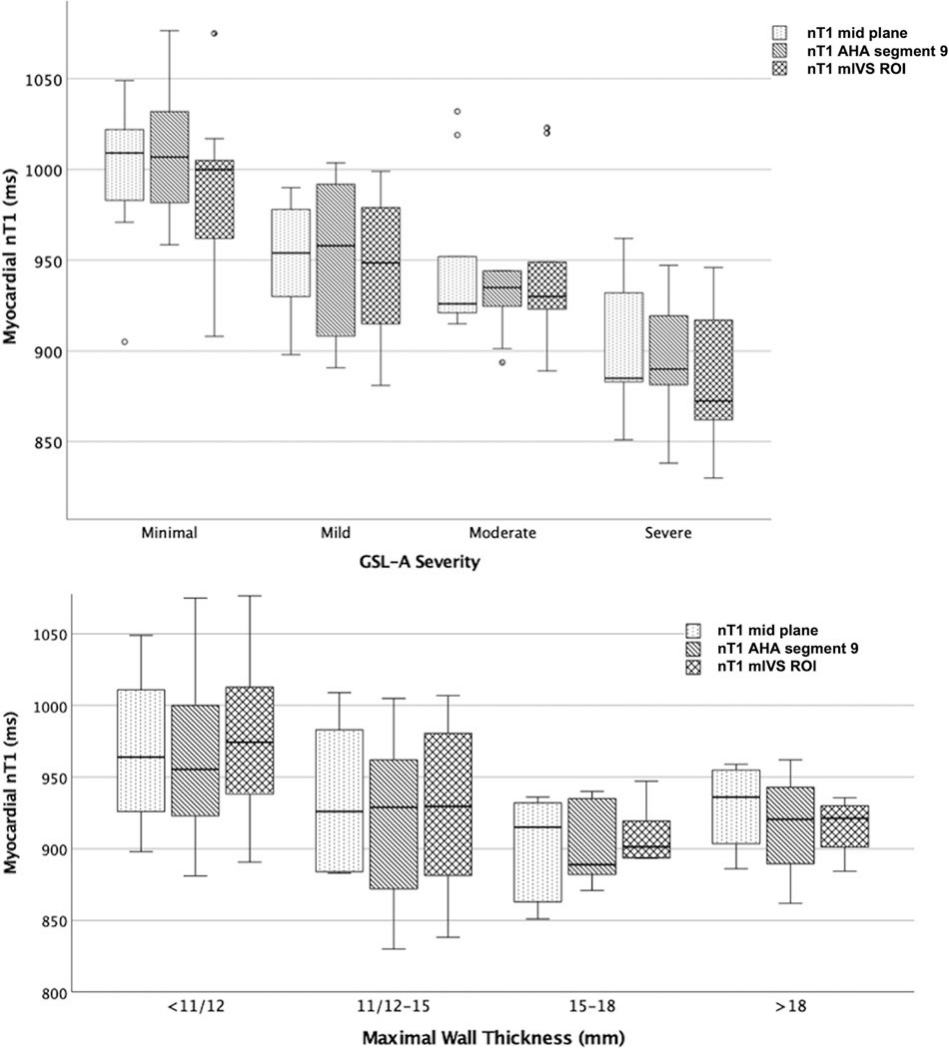
Box plot graphs of nT1 values measured at AHA segment 9, midventricular plane and mIVS ROI according to GSL-A and LVH severity. Distribution of nT1 values for different degrees of GSL-A (upper row) and LVH (lower row). In all the box plots, the top of the box represents the third quartile and the bottom the first quartile. The horizontal line represents the median for the entire cohort. The whiskers go from each quartile to the minimum or maximum

nT1 values according to GSL-A and LVH severity are depicted in Fig. 3.

Accordingly, both LVMM/BSA and MWT showed a moderate negative linear correlation with nT1 values in all measurement sites (for LVMM/BSA r = −0.451 to −0.614; *p* = < 0.001 to 0.003 and for MWT r = −0.368 to −0.511; *p* = < 0.001 to 0.02).

### Mapping and LGE features according to GSL-A degrees

Significant differences among groups were found in all nT1 values, as shown in Table 2.

At post hoc subgroup analysis, nT1 of AHA segment 9 and nT1 mid-plane were significantly different between groups 1 vs 2, 3 and 4 and between groups 4 vs 1, 2 and 3, while nT1 mIVS ROI was different only in patients from group 1, 2 and 3 when compared to subjects from group 4.

Regarding T2 values, the minimal and mild groups exhibit comparable readings across almost all measurement sites, while there is a rising tendency in the moderate-to-severe group. Moreover, when comparing group 4 to groups 1, 2, and 3, statistically significant differences were observed.

No significant differences were found in terms of ECV values among the four groups.

Areas of LGE were found in 17 patients (42.5%), mostly involving the infero-lateral wall (70.6%), while only one patient showed septal involvement with a diffuse LGE pattern. LGE distribution pattern was prevalently subendo-mesocardial (6/17, 35.3%) and mesocardial (5/17, 29.4%), with group 4 having significantly higher LGE values compared to the other groups, both in prevalence and as percentage of myocardial mass.

### Correlations between histopathological changes and CMR features

A moderate positive linear correlation was found between storage vacuoles accumulation and LVMM (r = 0.544; *p* < 0.001) and MWT (r = 0.470; *p* = 0.002), as shown in Supplementary data online, Fig. S3.

A significant negative correlation was found between storage vacuoles percentage and all nT1 values.

**Fig. 4:**
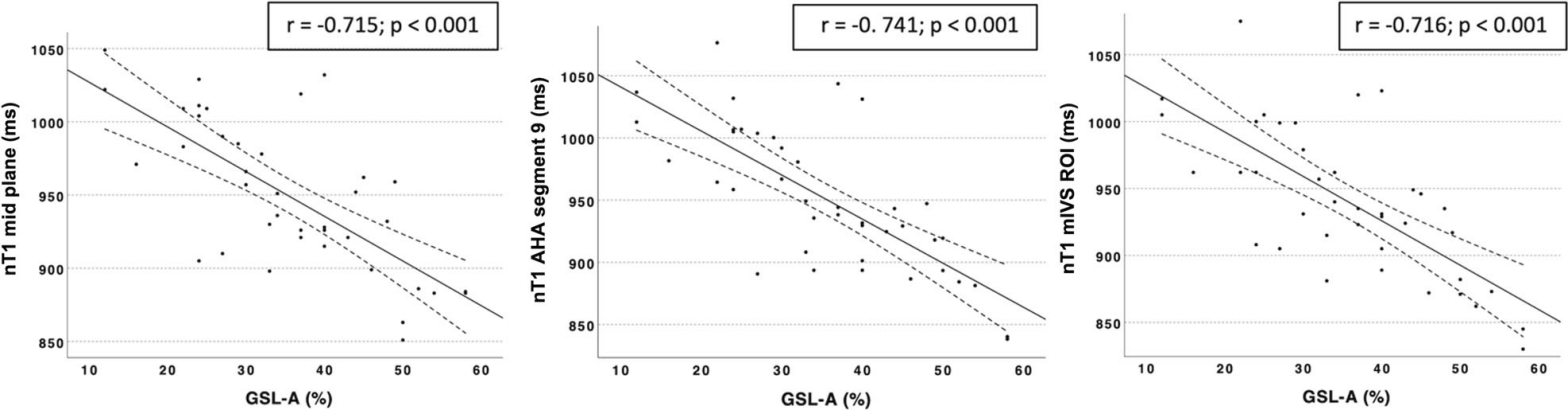
Correlation between nT1 values measured at midventricular plane, AHA segment 9, mIVS ROI vs GSL-A

The best correlation was found between nT1 AHA segment 9 (r = −0.741; *p* < 0.001), nT1 mIVS ROI (r = −0.716; *p* < 0.001) and mid-plane nT1 (r = −0.715; *p* < 0.001) (Fig. 4).

**Fig. 5:**
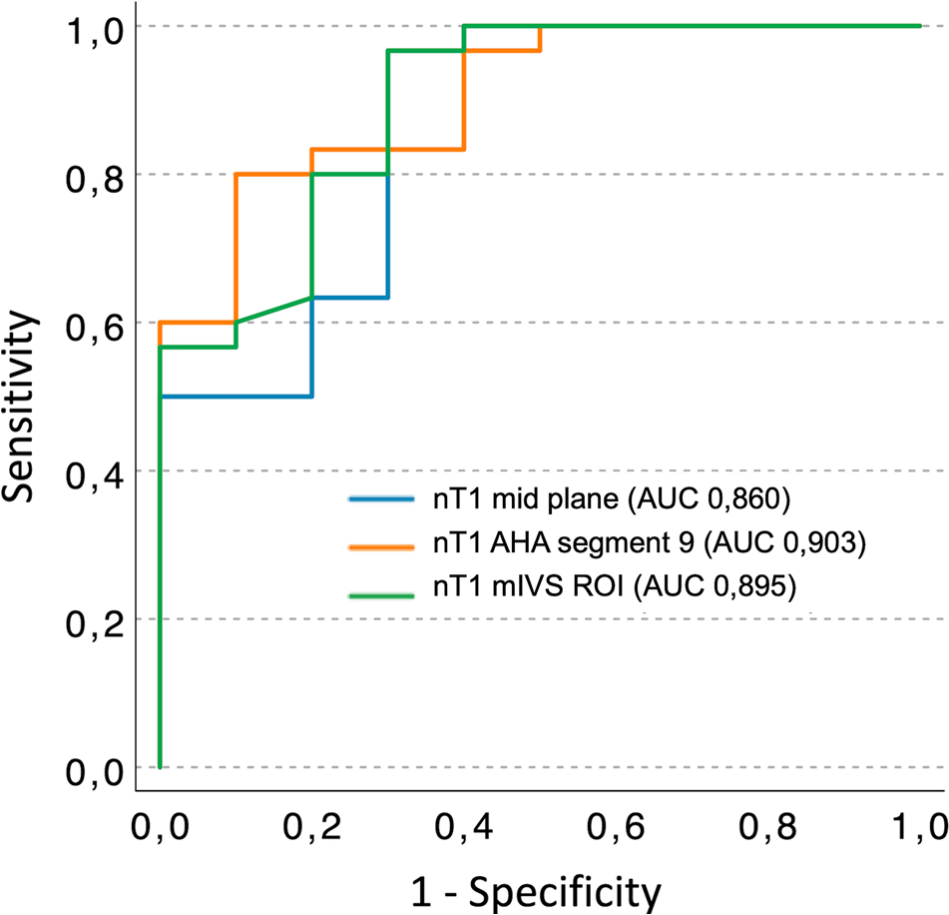
Diagnostic performance of nT1 values measured at midventricular plane, AHA segment 9, mIVS ROI in detecting patients with severe GSL-A. ROC curve illustrates the diagnostic performance of nT1 measured at the best three myocardial sites to identify patients with severe GSL-A

Therefore, nT1 of mid slice, mIVS ROI and AHA segment 9 were chosen to compute ROC curve analysis (Fig. 5), showing excellent diagnostic accuracy in discriminating group 4 patients from other groups (AUC = 0.860, 0.895, and 0.903, respectively).

nT1 of AHA segment 9 showed the best performance in identifying patients with severe GSL-A; thus, we established a nT1 of AHA segment 9 value < 893.5 ms as the best cutoff in identifying FD patients with a severe degree of GSL-A (sensitivity 97%, specificity 60%).

No significant correlation was found between the diameter of the cardiomyocyte, percentage of myocardial fibrosis and all CMR parameters.

## Discussion

In the last few decades, CMR has gained a key role in the diagnostic pathway of FD, particularly in the late hypertrophic stages of the disease [11], where it serves as a valuable, noninvasive alternative to EMB for diagnosing FD-CM [20].

Specifically, nT1 shortening emerged as a reliable hallmark of GSL-A in patients with LVH [9]. Building on this evidence, our study investigated how nT1 values vary across different degrees of histologically assessed GSL-A and identified the myocardial region that provides the most representative measurements using EMB as the reference standard.

### nT1 values according to GSL-A degrees

According to the assumption that an increase in cellular lipid content causes a progressive reduction in the myocardial nT1 values, we found significant differences in nT1 among GSL-A groups and linear negative correlations between nT1 values and GSL-A.

Decreasing nT1 values in all measurement sites have been observed as GSL-A increased, except for global nT1 and AHA segments 2 and 3 in group 3 versus group 2. Group 1 patients had near-normal mean nT1, while 4 of 10 showed reduced values in at least one measurement site. Conversely, all group 4 patients had nT1 below the normal range. This result confirms the recent finding by Ditaranto et al [21], who observed a decrease in nT1 values inversely proportional to the increase in percentage vacuolated myocyte area (as a marker of GSL-A).

Notably, 8/10 patients (80%) had abnormal nT1 in both groups 2 and 3, with no significant difference in mean nT1 values at all measurement sites between the two groups.

A possible explanation relies on the evidence that GSL-A is not the only determinant of nT1 value in FD patients.

Myocardial edema and interstitial fibrosis, generally associated with local nT1 increase, may occur during FD progression, balancing or mitigating the nT1 reduction induced by GSL-A [22]. Indeed, intracellular GSL storage in cardiomyocytes and release in the interstitial space may generate a proinflammatory response, partly mediated by the exposure of cardiomyocyte antigens [22]. Finally, chronic inflammation and cardiomyocyte death may result in interstitial or replacement fibrosis, as depicted by LGE areas [1, 23].

If the normal or near-normal nT1 values in group 1 can be attributed to the limited sensitivity of the nT1 technique for detecting minimal GSL-A [24], it may be hypothesized that the overlapping nT1 values between patients with mild and moderate GSL-A degrees could be due to the opposing effect of inflammatory changes on nT1. These changes are more pronounced in the group with moderate GSL-A.

**Fig. 6:**
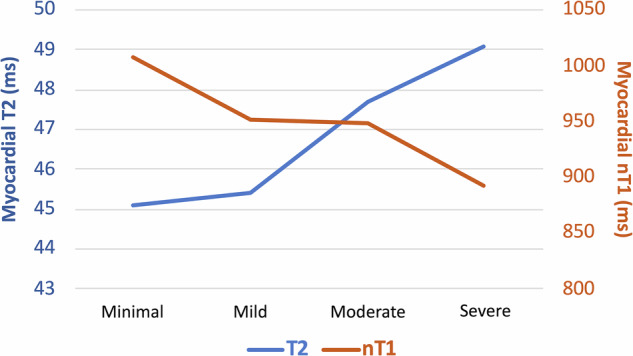
Line graph of mean nT1 and T2 values among the GSL-A groups. The graph shows a gradual decline in nT1 values along with growing GSL-A degree (orange line), with a plateau in mild-moderate groups, whereas matched T2 values show an opposite trend (blue line)

This hypothesis is supported by greater myocardial T2 values and a higher prevalence of CMR signs of edema in patients with moderate than mild GSL-A in our cohort. As shown in Fig. 6, the nT1 and T2 values have opposite trends, whereas the plateau of nT1 values in groups 3 and 4 is matched by the steeper slope of increasing T2.

This interplay between lipid accumulation (which lowers nT1) and superimposed inflammatory or edematous changes (which raise nT1) can mitigate the nT1 reduction observed in FD-CM, even though nT1 values generally remain below the normal range. This phenomenon, often referred to as ‘pseudonormalization,’ may therefore limit the ability of nT1 to accurately reflect the true degree of myocardial GSL-A [1, 24].

No significant differences in ECV values were observed among the study groups, which is consistent with the pathophysiology of FD, which is primarily an intracellular storage disorder during its early and intermediate phases, resulting in normal or near-normal ECV [24]. Only in advanced disease, when replacement fibrosis develops, does ECV tend to increase due to expansion of the extracellular matrix [11]. Although overall group comparisons were not significant, pairwise analyses showed higher ECV in the most severe GSL-A group (group 4) compared with groups 1 and 2, while intermediate groups showed no meaningful differences. This trend reinforces that native T1 is more sensitive to early intracellular lipid storage, whereas ECV primarily reflects extracellular remodeling and rises only in the later phases of disease.

### nT1 values according to LVH degree

Multiple studies reported that the myocardial nT1 decreases as the LVH increases, reflecting the gradual effects of long-standing cardiomyocytes GSL-A [9, 11, 24]. Accordingly, in our population, a higher prevalence of abnormal nT1 values was found in patients with mild (77.8%) and moderate-to-severe (100%) LVH when compared to patients with normal MWT (52.2%), and inverse correlations were found between LVH indexes (LVMM/BSA and MWT) and all nT1 values.

In our study, group 1 included patients with minimal GSL-A and normal MWT (pre-hypertrophic stage), which resulted in nT1 values within or bordering the normal range.

As reported, this stage of the disease is characterized by a paucity of symptoms and a gradual decrease in nT1, which remains in the normal range in most subjects [24].

Detecting GSL-A in the pre-hypertrophic stage of FD-CM represents the most critical diagnostic challenge, as emerging evidence suggests that the ERT is more efficient in preventing or limiting GSL-A before irreversible myocardial damage has been established [25, 26].

In previous reports [11, 15], the prevalence of pre-hypertrophic FD subjects with reduced nT1 values ranges from 41% to 44%, reaching 59% if an additional measurement site for nT1 was used. In our cohort, 14 out of 22 pre-hypertrophic FD patients had reduced nT1 values (63.6%) in at least one measurement site. The increased prevalence of nT1 value alteration in pre-hypertrophic FD patients may rely on a greater sensitivity conferred by the use of multiple measurement sites for these values.

### Diagnostic accuracy based on nT1 measurement site

Another major result of our study is the definition of the most performant site for nT1 measurement in FD patients for the GSL-A grading.

The protocol suggested by the Society for Cardiovascular Magnetic Resonance position paper [27] includes three nT1 maps acquired on basal and midventricular short-axis views and a single three-chamber view; nevertheless, no specific indications exist on the best nT1 measurement site. Commonly, the mid-wall septum is considered the most reliable nT1 measurement localization [28], due to the lesser influence of motion artifacts and susceptibility artifacts related to liver, lungs, and veins proximity [27]. However, there are no studies investigating which site may reflect more accurately the myocardial GSL-A.

Our results support the use of the midventricular septum as the preferential site for nT1 measurements, especially for serial assessments over time, given its close correlation with the presence of GSL-A, as validated histologically. Moreover, nT1 measured at these sites also improves the recognition of patients with a severe degree of GLS-A, which may be particularly useful for prognostic and therapeutic purposes, as advanced stages of the disease are associated with a greater incidence of CV events [2, 6].

Furthermore, recent studies suggested that, at a late stage of FD, ERT reduces symptoms without preventing disease progression toward organ loss and subsequently death [29–31]. Therefore, anticipating the ERT at earlier stages could be more beneficial [25].

The standardization of the measurement sites and the establishment of appropriate cutoffs are essential steps for the use of nT1 not only in diagnosing FD but also in stratifying disease severity.

### Limitations

This study has several limitations.

First, the monocentric retrospective design and small sample size limit the generalizability of our results. Indeed, the proposed nT1 cutoffs are site-specific, as mapping values vary with scanner hardware, field strength, sequence parameters, and individual factors such as age, sex, and patient phenotype. This variability restricts the universal applicability of a single threshold and underscores the need for multicenter validation and harmonized reference ranges.

Second, the high prevalence of pre-hypertrophic patients may affect the representativeness of our population and the categorization of the different grades of GSL-A.

Third, the size and the location of the EMB specimen could not be entirely representative of the global myocardial GSL-A; moreover, the strong correlation between GSL-A and nT1 values of mid-wall septal regions could be influenced by the site of EMB, which was performed in the septal myocardium. In our center, endomyocardial biopsies from other myocardial regions are not routinely performed due to the higher procedural risks; however, future studies including noninvasive inflammation markers or multimodal imaging approaches could further address this limitation.

Another limitation is the inclusion of female FD patients in our cohort, which may introduce variability related to the X-linked inheritance and mosaic expression patterns in women [24, 32]. Larger studies with broader patient populations will be better suited to explore sex-related differences in myocardial involvement in this disease.

Finally, the GSL-A at histology does not necessarily reflect the FD-CM severity, as the prognosis of FD patients may depend on multiple pathophysiological mechanisms [6].

## Conclusion

Myocardial nT1 value is strongly correlated with GSL-A, even though influenced by other concomitant phenomena such as inflammation and fibrosis. The midventricular septum is the measurement site with the best agreement with GSL-A at histology. The magnitude of nT1 values could help stratify FD patients, especially to identify those with severe GSL-A, which are at a more advanced stage of the disease.

## Supplementary information

ELECTRONIC SUPPLEMENTARY MATERIAL

## Data Availability

The datasets used and/or analyzed during the current study are available from the corresponding author on reasonable request.

## Abbreviations

AHA: American Heart Association
BSA: Body surface area
CM: Contrast media
CMR: Cardiac magnetic resonance
ECV: Extracellular volume
EMB: Endomyocardial biopsy
ERT: Enzyme replacement therapy
FD: Fabry disease
FD-CM: Fabry disease cardiomyopathy
GSL-A: Glycosphingolipid accumulation
ICC: Interclass correlation coefficient
LGE: Late gadolinium enhancement
LVH: Left ventricular hypertrophy
LVMM: Left ventricular myocardial mass
MWT: Maximum wall thickness
nT1: Native T1
ROC: Receiver operator characteristic
ROI: Region of interest
