# Supplementary material for: Diagnostic performance of native T1 in grading histologically proven myocardial glycosphingolipid accumulation in Fabry disease
Source: Insights Imaging. 2026 Apr 22;17:114. doi: 10.1186/s13244-026-02262-z (PMC13103178; doi:10.1186/s13244-026-02262-z)
Supplement: Supplementary file 1 — ELECTRONIC SUPPLEMENTARY MATERIAL [file 13244_2026_2262_MOESM1_ESM.pdf]

**Diagnostic performance of native T1 mapping in grading histologically-proven myocardial glycosphingolipid accumulation in Fabry Disease**

**ELECTRONIC SUPPLEMENTARY MATERIAL**

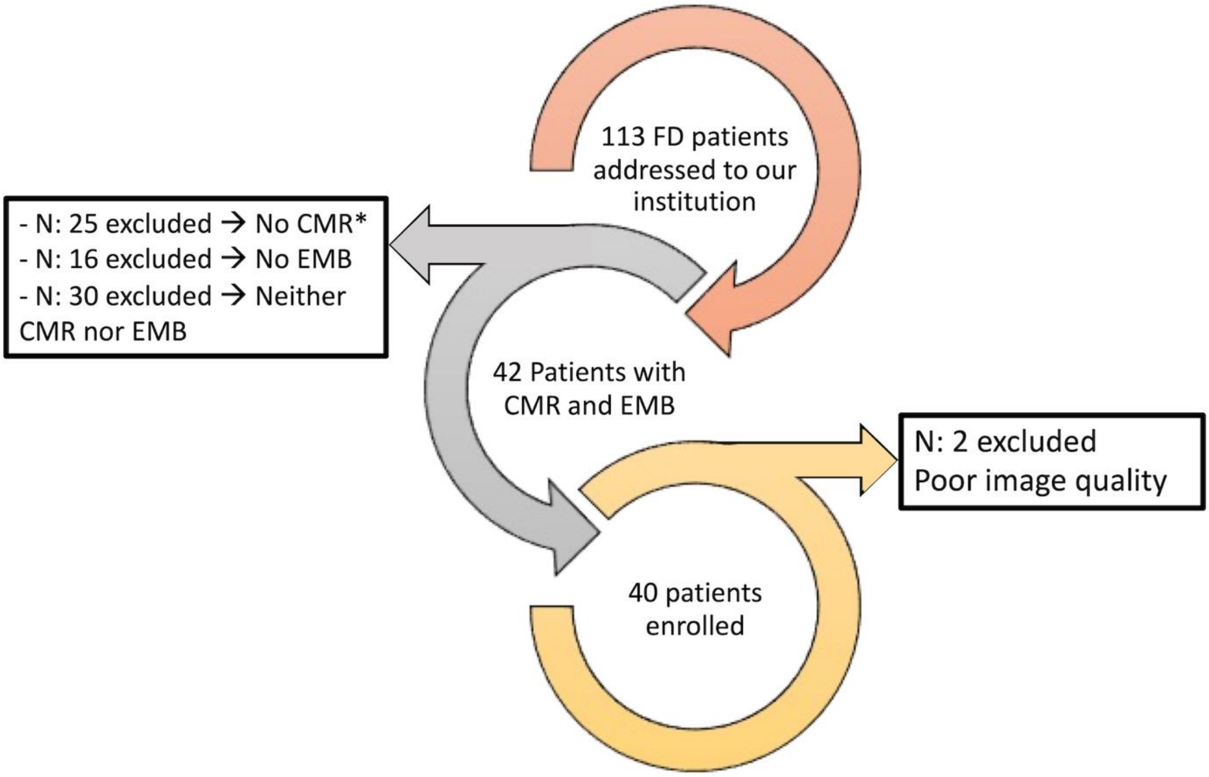


**Figure S1.** Patients' recruitment flow chart

*CMR: Cardiac Magnetic Resonance; EMB: Endomyocardial Biopsy*

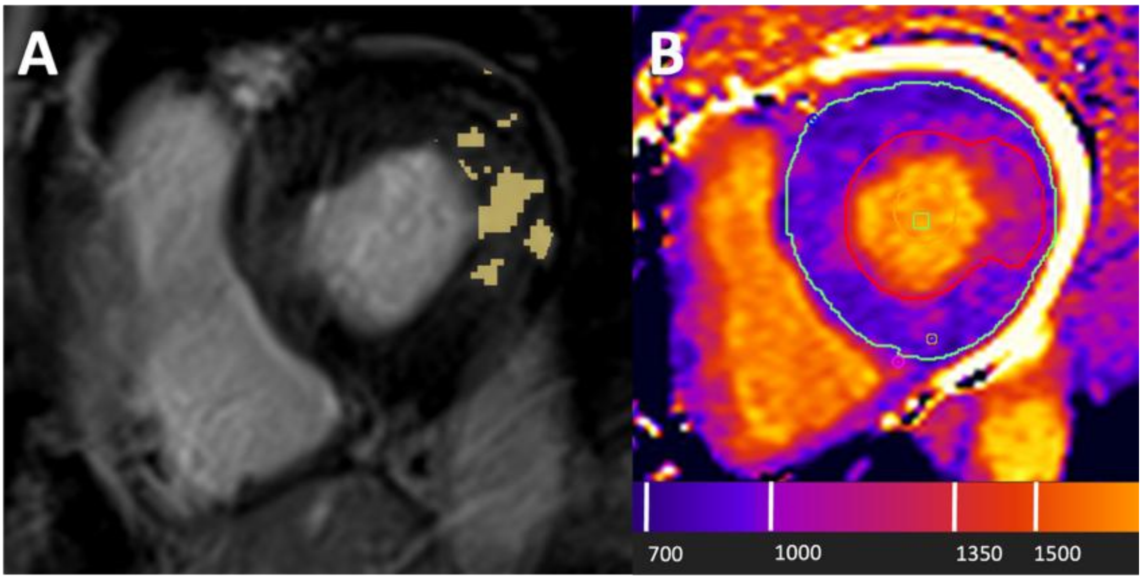


**Figure S2.** Contouring technique for measuring mapping values (B), by excluding LGE-positive regions (A)

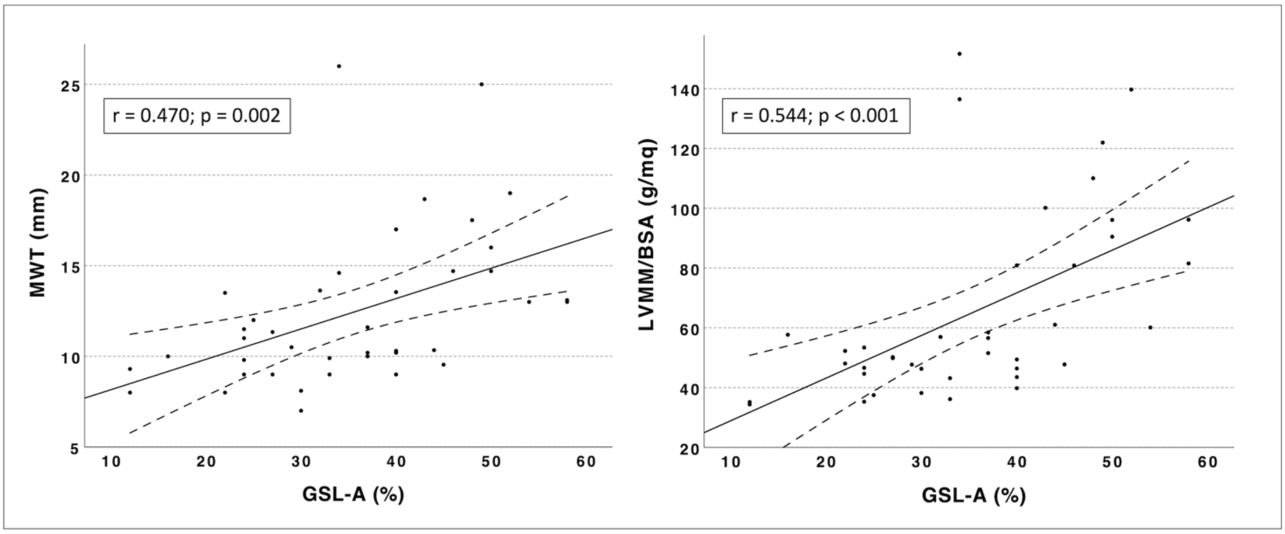


**Figure S3.** Correlation between MWT and LVMM/BSA vs GSL-A

MWT: maximum Wall Thickness; LVMM/BSA: Left Ventricle Myocardial Mass/Body Surface Area; GSL-A: glycosphingolipids Accumulation

SUPPLEMENTAL APPENDIX

# CMR sequence parameters and image reconstruction

- ***CineMR balanced-Steady state free-precession sequence (cineMR)***: TR 51,3, TE 1,21, a flip angle 45°, a 8 mm slice thickness, a matrix of 256x256, a field of view ranging from 340 mm to 400 mm and a voxel size of 2,0 x 1,3 x 8,0 mm. A stack of 10-12 contiguous short-axis (SA) slices completely encompassing both ventricles from the base to the apex was acquired, along with 1–3 contiguous slices for the 2-chamber (2ch), 3-chamber (3ch), and 4-chamber (4ch) views.

- ***Modified Look-Locker inversion recovery prototype or MOLLI sequence (T1 mapping)*** (package Siemens WIP #448) was acquired in three SA slices (basal, mid-ventricular, and mid-apical) and one 4ch slice, with following parameters: matrix 218 x 256, voxel size 1.41 x 1.41 x 8 mm3, TR/TE 2.6/1.12 ms, FA 35. For pre-contrast acquisitions the inversion pulse scheme was 3(3) 3(3)5 consisting of 3 inversions with 3 images after the first inversion, a 3-heartbeat pause, 3 inversions with 3 images after the second inversion and then the last 5 images. For post-contrast acquisitions, the protocol was 4(1)3(1)2 consisting of 4 images acquired after the first inversion pulse and a one-heartbeat pause for the complete recovery of magnetization. Then 3 and 2 images, respectively, were acquired after the second and third inversion, separated by a one-heartbeat pause. Acquired images were then processed applying a motion correction algorithm provided in the package, designed to adjust in-plane mis-registration between images that may be caused by diaphragm position drift or heartbeat irregularities. Two different sequence schemes were used for pre- and post-contrast acquisitions. The MOLLI source images were fitted with a three-parameter curve, with the automatic pixel-by-pixel generation of T1 map. In case of inadequate breath holding leading to translational displacement which can result in misregistration errors, position of the source images was initially manually adjusted. Extracellular volume fraction (ECV) map was then automatically generated by combining native and contrast-enhanced T1 map, inserting hematocrit value sampled before starting CMR exam, using the following formula:

$$ECV = (1 - hematocrit) \times (\Delta R1_{tissue} / \Delta R1_{blood})$$

where R1 = 1/T1 and the T1 values are given in milliseconds.

- ***<u>T2-prepared True-FISP prototype sequence (T2 mapping)</u>*** generated three different images with three different interval from T2 preparation pulse: 0ms, 24ms, and 55 ms (package Siemens WIP #448). Similarly to T1 mapping, acquired images were processed applying a motion correction algorithm, in three SA slices (basal, mid-ventricular, and mid-apical) and one 4ch slice.

- ***<u>Short tau inversion recovery T2 weighted sequence (T2w-STIR)</u>*** is a breath-hold black-blood segmented turbo spin echo sequence using a triple inversion recovery preparation module. A stack of 10-12 contiguous SA slices completely encompassing both ventricles from the base to the apex was acquired, , along with 1–3 contiguous slices for the 2ch and 4ch views. Sequence parameters were: TR 2 R-to-R intervals, TE 75 ms, flip angle 180°, TI 170 ms, slice thickness 8 mm, no interslice gap, field of view 340 to 400 mm, matrix 256 x 256, and a voxel size of 2.3 x 1.3 x 8 mm).

- ***<u>Contrast-enhanced inversion recovery phase-sensitive T1-weighted sequence (IR-CE T1w)</u>*** for late enhancement sequence was settled with the acquisition window to mid-end diastole and the following parameters: field-of-view: 380 – 400 mm, slice thickness: 8 mm, repetition/echo time: 4.6/1.3 ms, flip angle: 208, matrix: 256 × 192. Meticulous attention was paid to select the inversion time to suppress the signal of normal myocardium with typical values of 250–320 milliseconds. A stack of 10-12 contiguous SA slices completely encompassing both ventricles from the base to the apex was acquired, , along with 1–3 contiguous slices for the 2ch, 3ch, and 4ch views.

## Endomyocardial Biopsy Procedure Details

The endomyocardial samples were embedded in paraffin and fixed in buffered formalin at 10%. Hematoxylin and eosin, Masson trichrome, and Miller's Elastic Van Gieson were used to stain sections (5 μm). On frozen sections, histochemistry using the PAS and Sudan black stains was carried out to gauge the relative concentrations of intracellular glycosphingolipid deposits.

According to previous study (24), in situ ligation of hairpin probes with single-base 3′ overhangs was utilized to detect cardiomyocyte apoptosis (hairpin 1) and blunt DNA ends to measure necrosis (hairpin 2). Endomyocardial samples were produced and examined for transmission electron microscopy as previously mentioned (25). Through the use of a computerized, operator-guided system, myocardial fibrosis was measured (Advanced Research NIS Elements Imaging Software 4.30,

## Reference Cohort for myocardial nT1, T2 and ECV values

To derive scanner- and sequence-specific reference values for native T1, T2 and extracellular volume (ECV), we retrospectively assembled a control cohort of 100 individuals who had undergone clinical CMR at our institution and who showed no evidence of myocardial pathology on clinical, echocardiography and CMR imaging.

The cohort consisted of 50 females and 50 males, distributed across age ranges to reflect a broad adult population (20 controls for each age group: 20–30 yrs, 30–40 yrs, 40-50 yrs, 50-60 yrs, 60-70 yrs).

In all patients CMR examinations were performed for non-myocardial indications such as clarification of equivocal echocardiographic findings, evaluation of intracardiac pseudomasses, assessment of benign arrhythmias, exclusion of low-probability cardiomyopathies and characterization of congenital or anatomical variants. In all cases, ventricular morphology, function and tissue characterization were entirely normal.

All subjects met the following exclusion criteria:

- previous history of cardiovascular disease,
- known diabetes mellitus,
- uncontrolled hypertension,
- features suggestive of obstructive coronary artery disease,
- any CMR finding of myocardial edema, fibrosis, infiltration or functional abnormality.

| | Men (n: 50) | Women (n: 50) | Overall |
|---|---|---|---|
| Age (years) | 45.5 ± 13.6 | 46.5 ± 14.4 | 46.0 ± 14.0 |
| nT1 (ms) | 990.2 ± 12.5 | 1006.7 ± 10.7 | 998.5 ± 14.2 |
| T2 (ms) | 45.5 ± 1.4 | 47.1 ± 1.8 | 46.3 ± 1.8 |
| ECV (%) | 25.5 ± 2.2 | 25.9 ± 1.5 | 25.7 ± 1.9 |
| *Data are presented as mean ± standard deviation* | | | |

Table S1. Demographic characteristics and mapping parameters of the control cohort, presented overall and stratified by sex.
nT1: native T1; ECV: extracellular Volume
